# Pulmonary Arterial Capacitance Predicts Cardiac Events in Pulmonary Hypertension Due to Left Heart Disease

**DOI:** 10.1371/journal.pone.0165603

**Published:** 2016-11-22

**Authors:** Koichi Sugimoto, Akiomi Yoshihisa, Kazuhiko Nakazato, Yuichiro Jin, Satoshi Suzuki, Tetsuro Yokokawa, Tomofumi Misaka, Takayoshi Yamaki, Hiroyuki Kunii, Hitoshi Suzuki, Shu-ichi Saitoh, Yasuchika Takeishi

**Affiliations:** 1 Department of Cardiovascular Medicine, Fukushima Medical University, Fukushima, Japan; 2 Department of Pulmonary Hypertension, Fukushima Medical University, Fukushima, Japan; Scuola Superiore Sant'Anna, ITALY

## Abstract

**Background:**

Although pulmonary hypertension due to left heart disease (LHD-PH) accounts for the largest proportion of pulmonary hypertension, few reports on the epidemiological analysis of LHD-PH exist. Recently, pulmonary arterial capacitance (PAC) has attracted attention as a possible factor of right ventricular afterload along with pulmonary vascular resistance. We therefore investigated the clinical significance of PAC in LHD-PH.

**Methods:**

The subject consisted of 252 LHD-PH patients (145 men, mean age 63.4 ± 14.7 years) diagnosed by right heart catheterization. PAC was estimated by the ratio between stroke volume and pulmonary arterial pulse pressure. Patients were classified into four groups according to the PAC (1^st^ quartile was 0.74 to 1.76 ml/mmHg, the 2^nd^ quartile 1.77 to 2.53 ml/mmHg, the 3^rd^ quartile 2.54 to 3.59 ml/mmHg, and the 4^th^ quartile 3.61 to 12.14 ml/mmHg). The end-points were defined as rehospitalization due to worsening heart failure and/or cardiac death. The Cox proportional hazard regression model was used to determine what variables were associated with cardiac events.

**Results:**

The patients in the 1^st^ quartile had the lowest cardiac index and stroke volume index, and the highest mean pulmonary arterial pressure, mean pulmonary capillary wedge pressure, and pulmonary vascular resistance compared with the 2^nd^, 3^rd^, and 4^th^ quartiles. Fifty-four patients experienced cardiac events during the follow-up period (median 943 days). The event-free rate of the 1^st^ quartile was significantly lower than that of the 3^rd^ and 4^th^ quartiles (66.7% vs 82.5% [3^rd^ quartile], P = 0.008; and 92.1% [4^th^ quartile], P < 0.001). The Cox hazard analysis revealed that PAC was significantly associated with cardiac events (HR 0.556, 95% CI 0.424–0.730, P < 0.001).

**Conclusion:**

PAC is useful in the prediction of cardiac event risk in LHD-PH patients.

## Introduction

In pulmonary arterial hypertension (PAH), narrowing of the vessel lumen occurs by thickening of the medial wall or growth of endothelial cells in the peripheral vessels of the lung. Pulmonary vascular resistance (PVR) is defined as (mean pulmonary artery pressure [PAP]—mean pulmonary capillary wedge pressure [Pcw])/pulmonary blood flow [1]. PVR reflects the degree of peripheral vascular narrowing and is used as an indicator of PAH severity. PVR is also known to be a prognostic predictor in patients with chronic heart failure or pulmonary hypertension [2, 3]. When considering the right ventricular afterload, the beat resistance, which is the resistance against pulsatile blood flow, is as important as PVR, the static resistance [1]. In a recent study of idiopathic pulmonary artery hypertension (IPAH), pulmonary arterial capacitance (PAC), which reflects the beat resistance, was recognized as an indicator of right ventricular afterload [4].

Pulmonary hypertension due to left heart disease (LHD-PH) is most common in pulmonary hypertension. LHD-PH was defined as mean Pcw > 15 mmHg and mean PAP ≥ 25 mmHg at rest according to the European Society of Cardiology (ESC) criteria [5]. LHD-PH is classified into two types by its mechanism. One type is isolated post-capillary pulmonary hypertension (I-pcPH), which is caused by passive pressure propagation due to a rise in the LV filling pressure. The other is combined post-capillary pulmonary hypertension (C-pcPH), which is associated with a stenotic lesion in the pulmonary arterial side in addition to the rise in the LV filling pressure. Recently, the importance of PAC has been reported for the prognosis of chronic heart failure patients [6, 7]. However, the impact of PAC on the prognosis of LHD-PH is largely unknown. Here, we investigated the association between PAC and the prognosis of LHD-PH including both of I-pcPH and C-pcPH.

## Methods

### Study Subjects

**Fig 1:**
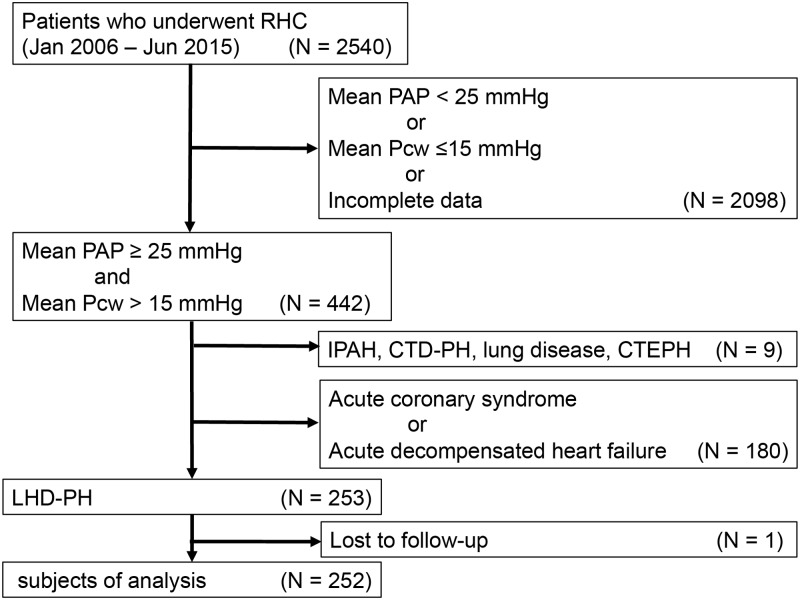
Algorithm of patient selection. RHC, right heart catheterization; PAP, pulmonary arterial pressure; Pcw, pulmonary capillary wedge pressure; IPAH, idiopathic pulmonary hypertension; CTD-PH, connective tissue disease-associated pulmonary hypertension; CTEPH, chronic thromboembolic pulmonary hypertension; LHD-PH, pulmonary hypertension due to left heart disease.

The study subjects consisted of consecutive 252 LHD-PH patients who had been diagnosed using right heart catheterization between January 2006 and June 2015 at Fukushima Medical University. We excluded cases of acute heart failure, acute coronary syndrome, IPAH, connective tissue disease-associated pulmonary hypertension, pulmonary hypertension due to lung disease, and chronic thromboembolic pulmonary hypertension. Fig 1 shows the enrollment criteria of the study subjects. Echocardiographic parameters and laboratory data were obtained from the medical records. Written informed consent was obtained from all study subjects. The study protocol was approved by the ethical committee of Fukushima Medical University in compliance with the Declaration of Helsinki.

### Hemodynamics Measurements

All catheterizations were performed within 7 days after echocardiography in a resting supine position under fluoroscopic guidance. PAP, Pcw, mean right atrial pressure (RAP) and cardiac output were measured using a 7F Swan-Ganz catheter (Edwards Lifesciences, Irvine, CA, USA). We mainly used the thermo dilution method for the measurement of cardiac output, however, for cases of advanced tricuspid regurgitation, we used the Fick method. The diastolic pressure gradient (DPG) was defined as the difference between the mean Pcw and the diastolic PAP [6]. The transpulmonary pressure gradient (TPG) was defined as the difference between the mean Pcw and the mean PAP [5]. PVR was calculated using the conventional formula. According to the two-element Windkessel model, the pulmonary artery pressure in diastole was assumed to show the exponential decay, and the PAC was estimated as the ratio between the stroke volume (SV) and the pulmonary arterial pulse pressure (PP) as reported previously [1, 4, 6, 7]. Patients were divided into four groups according to PAC for quartile analysis. The end-point was defined as cardiac death and/or rehospitalization due to worsening heart failure.

### Echocardiography

Transthoracic echocardiography was performed by an experienced echocardiographer using standard techniques [8]. The left atrial dimension, interventricular septal thickness, left ventricular (LV) end-diastolic diameter, LV end-systolic diameter, posterior wall thickness, LV end-diastolic volume, and LV ejection fraction (LVEF) were measured as echocardiographic parameters. All recordings were carried out on an ultrasound system (ACUSON Sequoia, Siemens Medical Solutions, Mountain View, CA, USA).

### Statistical Analysis

Normally distributed variables were presented as the mean ± SD, and non-normally distributed variables were presented as median (inter-quartile range). Categorical variables were expressed as numbers and percentages. The baseline characteristics of the groups were compared using analysis of variance for the continuous variables and the χ^2^ test for the non-continuous variables. Survival curves were estimated by the Kaplan-Meier method and compared using the log-rank test. Comparison of the area under the receiver-operating characteristic (ROC) curve (AUC) was carried out by a De Long test. A Cox proportional hazard model was used to analyze the association between clinical factors and cardiac events. The proportional hazards assumption for the model was checked by examining log minus-log transformed Kaplan-Meier estimates of the survival curves for two groups plotted against time to follow-up period. These curves help in identifying non-proportionality patterns in hazard function such as convergent (difference in risk between the 2 groups decreases with time), divergent, or crossing of the curves. In addition, Schoenfeld test for the violation of proportional hazards, which assess the correlation between scaled residuals and time, was also conducted. As the proportional-hazard assumptions were violated in the above-mentioned diagnostic test, the extended Cox hazard model was used for time-varying exposure of the adjusting variable. A P value of < 0.05 was considered significant for all comparisons. We considered the following clinical factors, which are generally known to affect the risk of cardiac events in heart failure patients: age, sex, atrial fibrillation, presence of ischemic etiology, New York Heart Association (NYHA) functional class, LVEF, levels of B-type natriuretic peptide (BNP), estimated glomerular filtration rate (eGFR), and hemoglobin. Additionally, the following hemodynamic parameters were selected based on previous studies: cardiac index, SV index, mean RAP, systolic PAP, diastolic PAP, mean PAP, mean Pcw, PP, PVR, DPG, and TPG [4, 6, 9]. All analyses were performed using SPSS version 21.0 (IBM, Armonk, NY, USA).

## Results

**Fig 2:**
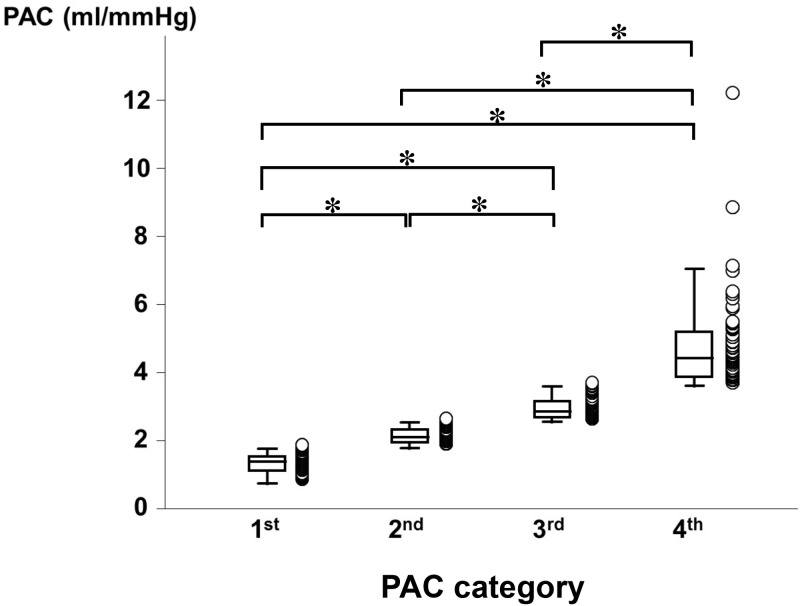
Distribution of PAC in each quartile. PAC, pulmonary arterial capacitance. * P<0.001.

**Fig 3:**
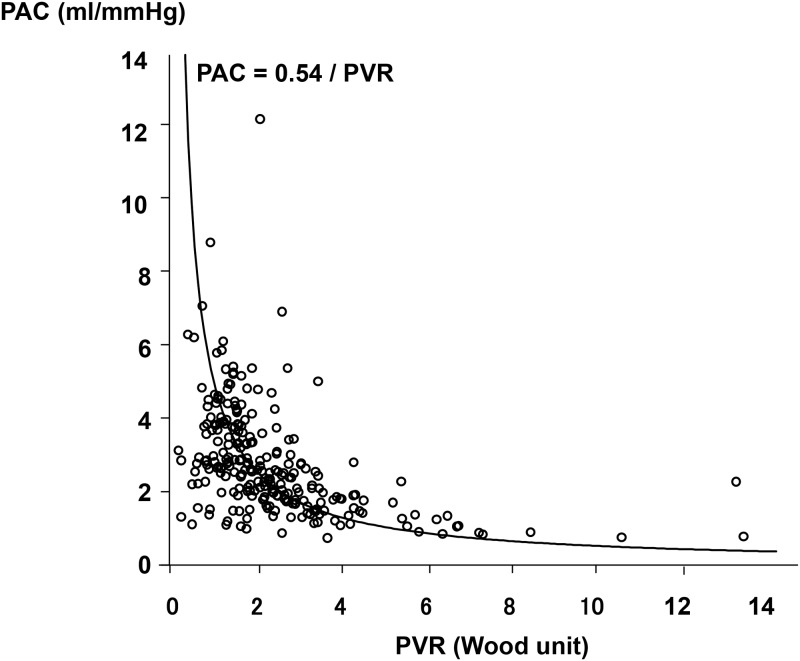
Scatterplot of PVR vs. PAC. There was a hyperbolic relationship between PAC and PVR. PVR, pulmonary vascular resistance; PAC, pulmonary arterial capacitance.

**Fig 4:**
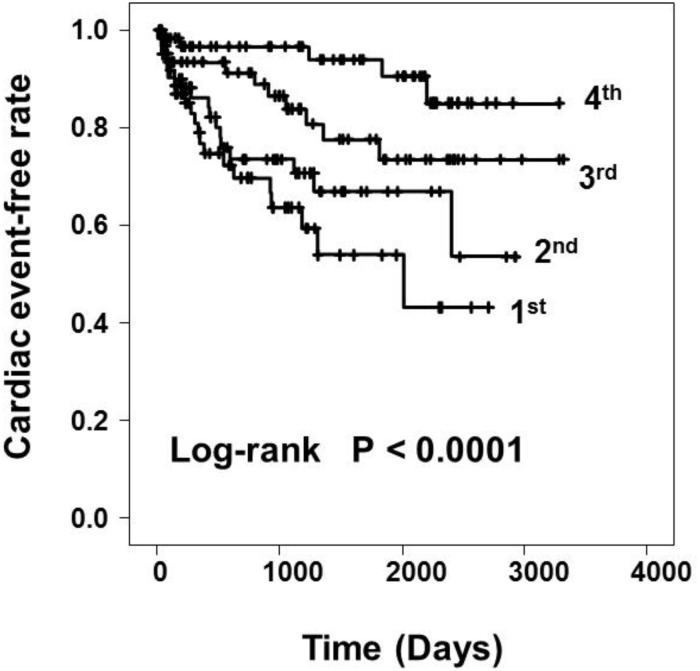
Kaplan-Meier curves for cardiac events according to quartiles of pulmonary arterial capacitance. Cumulative cardiac event-free rate was significantly lower in the 1^st^ quartile than the 2^nd^, 3^rd^, and 4^th^ quartiles.

In total, 253 patients were diagnosed with LHD-PH by right heart catheterization and 252 patients were followed-up (Fig 1). The median follow-up period was 943 days. A total of 54 patients (21.4%) were hospitalized due to worsening heart failure or cardiac death. Patients were divided into four groups, the 1^st^ to the 4^th^ quartiles, according to PAC. The range of PAC in the 1^st^ quartile was 0.74 to 1.76 ml/mmHg, the 2^nd^ quartile 1.77 to 2.53 ml/mmHg, the 3^rd^ quartile 2.54 to 3.59 ml/mmHg, and the 4^th^ quartile 3.61 to 12.14 ml/mmHg. Distribution of PAC in each quartiles was showed in Fig 2. As shown in Fig 3, there was a hyperbolic relationship between PAC and PVR. Table 1 shows the clinical characteristics, hemodynamic data, echocardiographic parameters, and laboratory data of each group. The 1^st^ quartile contained a larger number of patients with valvular heart disease and a higher NYHA class compared with the 4^th^ quartile. As for the hemodynamic parameters, the cardiac index (CI), SV index were significantly lower in 1^st^ quartile. The systolic PAP, diastolic PAP, mean PAP, mean Pcw, PVR, and TPG were significantly higher in the 1^st^ quartile. However, there were no significant differences in the levels of mean RAP and DPG. In the laboratory data, BNP was significantly higher in the 1^st^ quartile. In terms of medication for heart failure, the use of diuretics, digitalis, and inotropic agents was higher in the 1^st^ quartile. Fig 4 shows the Kaplan-Meier survival curves of the LHD-PH patients in each group. The 1^st^ quartile had the lowest event free rate compared to the other groups (66.7% vs. 73.0% [2^nd^ quartile], P = 0.276; 82.5% [3^rd^ quartile], P = 0.008; and 92.1% [4^th^ quartile], P < 0.001).

**Table 1:** Comparison of LHD-PH patient characteristics of each group.

Characteristic	1^st^ (n = 63)	2^nd^ (n = 63)	3^rd^ (n = 63)	4^th^ (n = 63)	P-value
Age (years)	65.3 ± 15.7	64.0 ± 13.8	61.1 ± 15.8	63.4 ± 14.7	0.449
Male (n, %)	31 (49.2)	35 (55.6)	37 (58.7)	42 (66.7)	0.992
Af (n, %)	31 (49.2)	34 (54.0)	29 (46.0)	40 (63.5)	0.222
HD (n, %)	9 (14.3)	2 (3.2)	9 (14.5)	11 (17.5)	0.080
Etiology of heart failure (n, %)					
Valvular heart disease (n, %)	29 (46.0)	23 (36.5)	19 (30.2)	6 (9.5) ** ††	<0.0001
Ischemic heart disease (n, %)	9 (14.5)	13 (20.6)	11 (17.5)	29 (46.0) ** †† §§	<0.0001
Cardiomyopathy (n, %)	5 (7.9)	10 (15.9)	11 (17.5)	7 (11.1)	0.366
Others (n, %)	20 (31.7)	18 (28.6)	22 (34.9)	21 (33.3)	0.888
NYHA classification (I/II/III/IV) (n)	(6/16/36/5)	(5/20/36/2)	(11/24/26/2)	(22/26/14/1) ** ††	<0.0001
Hemodynamic variables					
CI (l/min/m^2^)	2.1 ± 0.5	2.4 ± 0.5	2.9 ± 0.9 ** ††	3.0 ± 0.8 ** ††	<0.001
SVI (ml/m^2^)	26.5 ± 7.5	33.3 ± 7.6 **	42.2 ± 13.7 ** ††	46.7 ± 11.6 ** ††	<0.001
mean RAP (mmHg)	9.8 ± 4.9	10.9 ± 4.8	10.5 ± 4.9	10.4 ± 4.1	0.590
systolic PAP (mmHg)	57.3 ± 12.9	48.9 ± 8.7 **	44.7 ± 8.8 **	38.6 ± 5.4 ** †† §§	<0.001
diastolic PAP(mmHg)	25.6 ± 7.9	23.7 ± 6.2	21.0 ± 4.3 **	20.1 ± 4.2 ** †	<0.001
mean PAP (mmHg)	37.9 ± 8.7	34.1 ± 9.2 *	30.3 ± 5.8 ** †	27.9 ± 3.2 ** ††	<0.001
mean Pcw (mmHg)	25.7 ± 6.6	23.6 ± 5.6	21.7 ± 5.0 *	20.0 ± 3.2 ** ††	<0.001
PP (mmHg)	31.6 ± 9.4	25.3 ± 5.6 **	23.7 ± 7.4 **	18.0 ± 5.2 ** †† §§	<0.001
PVR (Wood unit)	4.0 ± 2.4	2.8 ± 1.7	1.8 ± 0.8	1.5 ± 0.6 ** ††	<0.001
PAC (ml/mmHg)	1.4 (0.4)	2.1 (0.4) **	2.8 (0.5) ** ††	4.4 (1.3) ** †† §§	<0.001
DPG (mmHg)	2.5 ± 3.9	1.4 ± 2.2	1.3 ± 2.6	1.5 ± 2.6	0.084
TPG (mmHg)	12.2 ± 6.9	10.5 ± 7.8	8.6 ± 4.3 **	7.9 ± 2.7 **	<0.001
systolic AoP (mmHg)	117.8 ± 27.8	126.9 ± 23.5	134.2 ± 32.3	143.0 ± 32.9 ** †	<0.001
diastolic AoP (mmHg)	67.7 ± 13.5	71.4 ± 12.4	68.7 ± 13.9	74.5 ± 15.6	0.067
mean AoP (mmHg)	86.4 ± 19.1	91.3 ± 13.1	93.7 ± 20.0	101.1 ± 18.1 ** †	0.001
Laboratory data					
BNP (pg/dl)	1521.8 ± 2045.6	679.6 ± 598.0 **	719.4 ± 883.2 **	420.0 ± 559.1 **	<0.001
eGFR (ml/min/1.73 cm^2^)	49.1 ± 27.0	54.5 ± 22.2	55.2 ± 33.6	51.6 ± 27.8	0.477
HbA1c (%)	5.8 ± 1.1	5.8 ± 0.7	5.7 ± 0.7	5.8 ± 1.3	0.406
UA (mg/dl)	6.8 ± 2.7	7.5 ± 2.3	6.5 ± 1.6	6.5 ± 1.6	0.692
Hemoglobin (g/dl)	12.2 ± 2.1	12.9 ± 2.7	12.6 ± 2.6	12.7 ± 2.4	0.412
Echocardiographic data					
LAD (mm)	43.4 ± 14.2	44.0 ± 13.0	37.9 ± 13.6	37.0 ± 12.1	0.126
IVS (mm)	10.9 ± 2.9	10.8 ± 2.9	11.2 ± 3.4	11.8 ± 2.4	0.258
LVDd (mm)	55.0 ± 11.7	55.5 ± 10.1	51.4 ± 11.4	52.5 ± 9.4	0.097
LVDs (mm)	43.4 ± 14.2	44.0 ± 13.0	37.9 ± 13.6	37.0 ± 12.1†	0.004
LVPW (mm)	10.9 ± 2.4	11.2 ± 2.7	11.8 ± 3.9	11.5 ± 2.1	0.322
LVEDV (ml)	136.1 ± 79.5	134.86 ± 60.7	118.01± 60.6	125.2± 67.1	0.405
LVEF (%)	43.4± 18.2	45.4± 18.3	48.2± 15.4	53.2± 14.8 *	0.009
Medical therapy					
Beta-blockers	49 (77.8)	52 (82.5)	44 (69.8)	42 (66.7)	0.157
ACE-inhibitor/ARB	51 (81.0)	58 (92.1)	52 (82.5)	48 (76.2)	0.116
Diuretics	46 (73.0)	43 (68.3)	40 (63.5)	25 (39.7) ** † §	0.001
Digitalis	13 (20.6)	9 (14.3)	7 (11.1)	2 (3.2) *	0.026
Inotropic agent	20 (31.7)	8 (12.7) *	7 (11.1) **	2 (3.2) **	<0.001

Values are shown as mean ± SD or median (inter-quartile range) or n (%). Af, atrial fibrillation; HD, hemodialysis; NYHA, New York Heart Association; CI, cardiac index; SVI, stroke volume index; RAP, right atrial pressure; PAP, pulmonary arterial pressure; Pcw, pulmonary capillary wedge pressure; PP, pulmonary arterial pulse pressure; PVR, pulmonary vascular resistance; PAC, pulmonary arterial capacitance; DPG, diastolic pressure gradient; TPG, transpulmonary pressure gradient; AoP, arterial pressure; BNP, brain natriuretic peptides; eGFR, estimated glomerular filtration rate; HbA1c, hemoglobin A1c; UA, uric acid; LAD, left atrial diameter; IVS, interventricular septal wall thickness; LVDd, left ventricular end-diastolic diameter; LVDs, left ventricular end-systolic diameter; LVPW, left ventricular posterior wall; LVEDV, left ventricular end-diastolic volume; LVEF, left ventricular ejection fraction; ACE-inhibitor, angiotensin converting enzyme-inhibitor; ARB, angiotensin receptor blocker.

* P<0.05 and ** P<0.01 vs. 1^st^ quartile, † P<0.05 and †† P<0.01 vs. 2^nd^ quartile, § P<0.05 and §§ P<0.01 vs. 3^rd^ quartile.

In the Cox hazard model, PAC, NYHA classification, non-ischemic etiology, SV index, mean RAP, systolic PAP, diastolic PAP, mean PAP, mean Pcw, PP, LVEF, BNP, eGFR, and hemoglobin level were significantly associated with cardiac events (Table 2).

**Table 2:** Predictors of cardiac events by Cox proportional hazards model.

Variables	Univariate		
	β coefficient	HR (95% CI)	P-value
Age	0.005	1.005 (0.987–1.025)	0.577
Male	-0.314	0.730 (0.417–1.278)	0.271
Af	0.211	1.234 (0.707–2.156)	0.460
IHD	-0.808	0.446 (0.217–0.917)	<0.001
NYHA classification	1.018	2.769 (1.854–4.136)	<0.001
CI	-0.277	0.758 (0.520–1.105)	0.150
SVI	-0.030	0.970 (0.949–0.992)	0.007
mean RAP	0.089	1.093 (1.037–1.152)	0.001
systolic PAP	0.032	1.003 (1.012–1.054)	0.002
diastolic PAP	0.060	1.062 (1.022–1.103)	0.002
mean PAP	0.031	1.089 (1.034–1.145)	0.001
mean Pcw	0.069	1.126 (1.056–1.200)	<0.001
PP	0.030	1.031 (1.001–1.062)	0.045
PVR	0.102	1.108 (0.992–1.237)	0.070
PAC	-0.587	0.556 (0.424–0.730)	<0.001
DPG	0.032	1.032 (0.944–1.129)	0.484
TPG	0.004	1.004 (0.964–1.046)	0.836
LVEF	-0.023	0.977 (0.961–0.992)	0.004
log BNP	1.222	3.394 (2.007–5.740)	<0.001
eGFR	-0.021	0.979 (0.970–0.989)	<0.001
Hemoglobin	-0.222	0.801 (0.719–0.892)	<0.001

Abbreviations as in Table 1.

**Fig 5:**
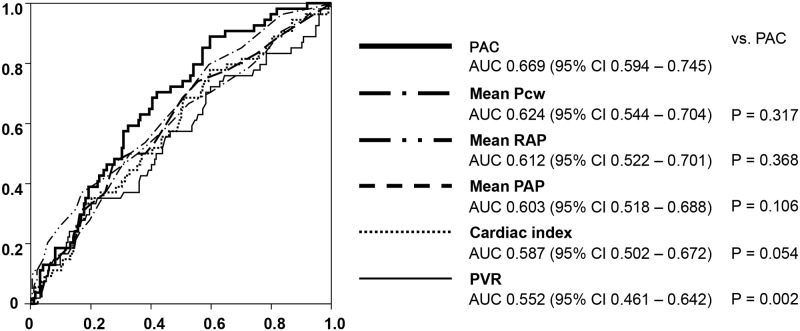
Receiver-operating characteristic (ROC) curve for PAC for prediction of cardiac events in LHD-PH patients. AUC, area under the curve; CI, confidence interval. PAC, pulmonary arterial capacitance Pcw, pulmonary capillary wedge pressure; RAP, right atrial pressure; PAP, pulmonary arterial pressure; PVR, pulmonary vascular resistance; LHD-PH, pulmonary hypertension due to left heart disease. Comparison of the AUC was carried out by a De Long test.

We analyzed the ROC curve of each hemodynamic index as a prognostic predictor of cardiac events and compared PAC with each hemodynamic index (Fig 5). The AUC of the PAC was 0.669 (95% confidence interval, 0.594 to 0.795), which was the highest in the hemodynamic indices. Compared to the PVR, PAC was a significantly superior predictor (AUC; 0.669 vs. 0.552, P = 0.002) as revealed by the De Long test.

**Fig 6:**
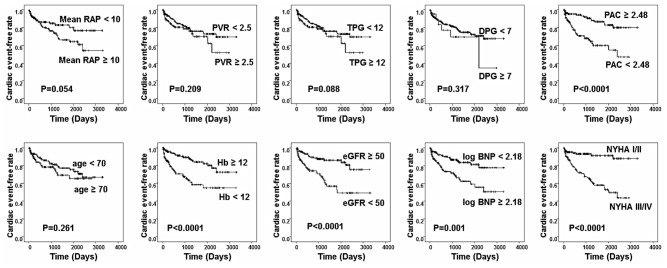
Kaplan-Meier curves for cardiac event-free rates in LHD-PH patients. The cardiac event-free rate significantly differed between PAC ≥ 2.48 and < 2.48 ml/mmHg, log BNP ≥ 2.18 and < 2.18, eGFR ≥ 50 and < 50 ml/min/1.73 cm^2^, Hemoglobin ≥ 12 and < 12 mg/ml, and NYHA classification III/IV and I/II. To the contrary, no difference was observed in the cardiac event-free rate between mean RAP ≥ 10 and < 10 mmHg, PVR ≥ 2.5 and < 2.5 WU, TPG ≥ 12 and < 12 mmHg, DPG ≥ 7 and < 7 mmHg, and age ≥ 70 and < 70 years.

Fig 6 shows the results of the Kaplan-Meier curves in each of the sub-group. We chose PAC of 2.48 ml/mmHg as cut-off value since it was the most balanced of the sensitivity and specificity from the result of ROC curve (the sensitivity was 70.4% and the specificity was 58.1% for the prediction of cardiac events). The low PAC group showed a significantly lower cardiac event-free rate compared to the high PAC group, whereas no differences in cardiac event-free rates were observed between the high and low groups for age, mean RAP, PVR, TPG, and DPG. Patients with NYHA classifications III or IV showed significantly lower cardiac event-free rates. In the biochemical data, patients with high levels of BNP and low levels of eGFR and hemoglobin also showed significantly lower cardiac event-free rates.

## Discussion

In the present study, we showed that patients with lower PAC had significantly higher cardiac event rates than those with higher PAC. PAC was significantly associated with cardiac death and worsening heart failure in LHD-PH patients.

Our study indicated that NYHA classification, BNP, anemia and CKD were recognized as predictive factors for cardiac events even if the patients were limited to LHD-PH. One of the possible mechanisms might be the involvement of cardio-renal-anemia syndrome in pulmonary hypertension as reported previously [10]. Yambe et al. reported that NYHA classification, eGFR, and hemoglobin level were better than the hemodynamic indices as predictive factors for cardiac death in LHD-PH [11]. However, their analysis did not include PAC. Since pulmonary blood flow is pulsatile, and not a continuous wave, it is insufficient to consider only PVR as the afterload of the right ventricle [1, 12]. Pellegrini et al. showed that the predictive ability of PAC for heart failure was retained in normal PVR patients with chronic heart failure, including patients without pulmonary hypertension [7]. Although the backgrounds of patients were different, our results were consistent with theirs at the point that PAC was a better predictive factor than PVR. Moreover, Stevens et al. reported that PA stiffness was an independent predictive factor of right ventricular failure in pulmonary hypertension from the cardiac MRI analysis [13]. The reason for the excellent predictive ability of PAC was considered to be that PAC reflected the influence of both PVR and Pcw [12]. The optimal cut-off value of PAC to predict cardiac events has not yet been determined. Depending on the patient’s background, various cut-off values have been suggested in several reports, including the present study [7, 9].

C-pcPH is known to be associated with a worse prognosis than I-pcPH [3, 6]. However, other studies have reported no difference between C-pcPH and I-pcPH in clinical outcome [14, 15]. Such discrepancies might be due to a small absolute value of DPG, which could easily be the cause of a measurement error [11]. However, the ESC guidelines have recently recommended the use of DPG, rather than TPG, for distinction of C-pcPH or I-pcPH (C-pcPH is defined as PVR > 3 Wood unit and/or DPG ≥ 7 mmHg) [5]. Al-Naamani et al. reported that PAC was the best predictor of mortality whereas DPG was not a prognostic predictor in heart failure with preserved EF [9]. Although the concept of dividing into I-pcPH and C-pcPH is important, their and our results suggested that the predictive ability of DPG for cardiac events was still unclear, and PAC seems to be the most powerful predictive factor of such events.

### Study Limitations

There are some potential limitations in the present study. First, we measured cardiac output by both thermo dilution method and Fick’s method. It raises the issue that there is a variability between Fick’s method and thermos dilution method. However, we used thermos dilution method in routine and the number of patients who used Fick’s method was small. Second, during this study period, volume analysis by cardiac MRI and the measurement of impedance were not performed in routine. Further investigation including these parameters is required in the future.

## Conclusion

In the present study, we showed the importance of PAC when predicting cardiac event risk in LHD-PH patients. PAC should also be considered as an important contributing factor of right ventricular afterload. Further multicenter studies are required in future to fully validate these findings.
